# Mediation analysis of depressive symptoms in the relationship between pulmonary function (measured by peak expiratory flow) and cognitive function among older adults in Chinese

**DOI:** 10.1371/journal.pone.0328231

**Published:** 2025-07-24

**Authors:** Chui-Yu Li, Hui-Rong Hu, Xiao-Qi Zhang, Yu-Mei Lin, Zhi-Yuan Chen

**Affiliations:** Department of Anesthesiology, the Second Affiliated Hospital of Fujian Medical University, Quanzhou, Fujian Province, China; UTRGV: The University of Texas Rio Grande Valley, UNITED STATES OF AMERICA

## Abstract

**Background:**

Population aging has led to accelerated decline in pulmonary function, rising prevalence of depressive symptoms, and cognitive impairment, posing significant public health challenges. Although epidemiological evidence links poorer pulmonary function to subsequent cognitive decline, the pathways driving this relationship are not fully understood. We therefore investigated whether depressive symptoms mediate this relationship in older Chinese adults.

**Methods:**

Data were drawn from the China Health and Retirement Longitudinal Study (CHARLS), including 2,320 participants aged 60 years and older. Pulmonary function was measured using peak expiratory flow (PEF). Depressive symptoms were assessed with the 10-item Center for Epidemiologic Studies Depression Scale (CES-D-10). Cognitive function was evaluated through tests of episodic memory and mental intactness. Mediation analysis was conducted using Baron and Kenny’s framework, and bootstrap analysis with 1,000 resamples was performed to assess direct and indirect effects. Sensitivity analyses ensured result robustness.

**Results:**

Spearman correlation analysis showed that PEF was positively correlated with cognitive function (r = 0.20, P < 0.001) and negatively correlated with depressive symptoms (r = −0.15, P < 0.001). After adjusting for confounders, multivariate linear regression analysis indicated that baseline PEF was positively associated with subsequent cognitive function (β = 0.131, P < 0.001) and negatively associated with depressive symptoms (β = −0.064, P < 0.001). The mediation effect of depressive symptoms accounted for 9.1% of the total effect regarding baseline pulmonary function on cognitive function.

**Conclusion:**

Depressive symptoms partially mediate the relationship between pulmonary function and cognitive function in older adults. These findings emphasize the need for mental health interventions to mitigate cognitive decline linked to reduced pulmonary function.

## 1. Introduction

Population aging has become a critical issue in China and worldwide. By 2050, there will be more than 500 million people in China who are 60 years of age or older, making up about 38.81% of the country’s total population [1]. The issue of population aging has drawn widespread attention, particularly the decline in lung function, rising prevalence of depression, and impaired cognitive function. These factors pose severe risks to the physical and psychological health of older populations.

Epidemiological studies in China and abroad report that reduced pulmonary function predicts subsequent cognitive decline [2–4]. Proposed mechanisms include chronic hypoxaemia, systemic inflammation, and microvascular damage, each of which can impair cerebral perfusion and neuronal integrity. However, the mechanisms underlying this association remain unclear because prior work has largely focused on direct effects and neglected potential psychological mediators [5].

Depressive symptoms seem to be a credible but underexamined mediator. Impaired lung function increases dyspnoea, limits physical and social activity, and is biologically linked to neuroinflammation and neurotransmitter imbalance—all of which heighten the risk of depression [6–8]. In our earlier Mendelian-randomisation work, chronic obstructive pulmonary disease (COPD)—a prototypical disorder of impaired lung function—causally increased the likelihood of depression [9]. Depression, in turn, is a well-established risk factor for accelerated cognitive decline via sustained inflammatory signaling, hypothalamic–pituitary–adrenal dysregulation, and reduced neuroplasticity [10,11]. However, no large‐scale longitudinal study has formally tested whether depressive symptoms mediate the lung–cognition link in Chinese older adults. Existing Chinese research is either cross-sectional, region-specific, or focuses solely on direct effects, leaving the mediation pathway unaddressed [5].

To address this gap, this study aims to utilize the China Health and Retirement Longitudinal Study (CHARLS) to systematically investigate the mediating effect of depressive symptoms on the relationship between pulmonary function and cognitive function.

## 2. Methods

### 2.1 Sample and study design

**Fig 1:**
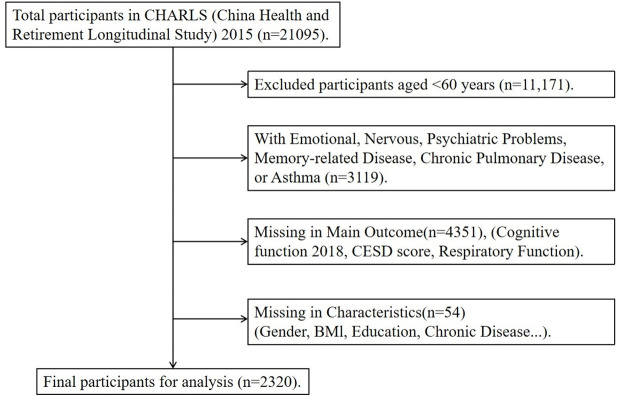
Participant inclusion ﬂow chart.

The data used in this study were derived from CHARLS, a nationwide longitudinal survey designed to assess health status and demographic characteristics of Chinese population aged 45 and older [12]. CHARLS adopts a multi-stage probability sampling method, and full details have been published elsewhere [13]. In this study, we analyzed data from the 2015 wave of CHARLS, which included approximately 22,000 participants from 450 villages across 150 counties in China. For this study, only participants aged 60 or older were included, and those previously diagnosed with psychiatric illnesses, memory impairment, chronic respiratory conditions, or asthma were excluded. After excluding cases with missing or abnormal values for essential variables, a final sample of 2320 participants were included in our analysis (Fig 1).

### 2.2 Measurements

#### 2.2.1 Cognitive function.

Cognitive function was assessed in the 2018 follow-up survey using two validated domains: episodic memory and mental intactness [14]. To measure episodic memory, interviewers first read a set of ten words aloud to participants, who were immediately asked to recall as many words as possible (immediate recall). Ten minutes later, participants were again asked to recall the same words (delayed recall). The episodic memory score was calculated as the average of immediate and delayed recall scores, ranging from 0 to 10 points [15].

Mental intactness was evaluated using tasks such as drawing a pentagon, correctly stating the present date, season, and weekday, as well as performing sequential subtractions by seven starting from 100. Each correct response contributed one point, with a maximum achievable score of 11 points. A global cognition index (range 0–21) was then derived by summing the mental-intactness and episodic-memory scores; higher values indicate better cognitive performance [8]. Consistent with earlier research, cognitive impairment was defined as a score ≤1 SD below the cohort mean [16].

#### 2.2.2 Depressive symptoms.

Depressive symptoms were assessed using the 10-item Center for Epidemiologic Studies Depression Scale (CES-D-10) [17]. This instrument evaluates participants’ emotional states experienced over the past week. Each item was rated on a four-point scale (0–3), with responses classified as follows: 0 indicated “rarely or none of the time (less than 1 day)”; 1 represented “some or a little of the time (1–2 days)”; 2 corresponded to “occasionally or a moderate amount of time (3–4 days)”; and 3 signified “most or all of the time (5–7 days)”. Total scores (sum of all items) range from 0 to 30, with higher scores indicating more severe depressive symptoms.

#### 2.2.3 Pulmonary function.

Pulmonary function was assessed by measuring peak expiratory flow (PEF) with a peak-flow meter under a standardized protocol to ensure consistency and accuracy. PEF is widely used as an index of ventilatory function; it shows a positive correlation with forced expiratory volume in the first second (FEV1) [18], and reflects airway patency, large-airway status, and respiratory muscle performance [19]. Participants were asked to avoid vigorous exercise, smoking, and caffeine for at least 30 minutes before the measurement. Each participant performed three maximal exhalations following a full inhalation; the mean of the three PEF readings was recorded as the final PEF value [20].

#### 2.2.4 Covariates.

In this study, demographic information, lifestyle behaviors, and health status were included as covariates to control for potential confounding effects in the relationships among the independent variable, dependent variable, and mediator. The covariates included age, gender, highest education level (categorized as illiterate, primary school, middle school, high school and above), marital status (single/married), place of residence, body mass index (BMI), smoking history, drinking history, total household wealth, physical activity (yes/no), and number of chronic conditions (0, 1, or ≥2). BMI was calculated from self-reported height and weight, then categorized as underweight (BMI < 18.5 kg/m²), normal weight (18.5 ≤ BMI < 25 kg/m²), overweight (25 ≤ BMI < 30 kg/m²), or obesity (BMI ≥ 30 kg/m²). All covariates were collected using standardized instruments to ensure consistency and reliability in the data.

### 2.3 Statistical analysis

Initially, continuous variables were described using means (standard deviation, SD), and categorical variables were presented as frequencies (percentages) to report the overall sample characteristics. Subsequently, Spearman correlation analysis was conducted to assess the relationships among PEF, depressive symptoms, and cognitive function. We assessed mediation of baseline PEF on follow-up cognitive function by depressive symptoms using a nonparametric bootstrap (1,000 resamples) in accordance with Hayes [21]. This approach yields robust indirect-effect estimates without normality assumptions. We reported the total, direct, and indirect effects, along with 95% confidence intervals. For conceptual clarity and comparison with prior literature, we also followed the traditional steps outlined by Baron and Kenny (1986) [22], using linear regression models to examine (1) the association between PEF and depressive symptoms, (2) the association between depressive symptoms and cognitive outcomes, and (3) the association between PEF and cognitive outcomes controlling for depressive symptoms. Regression results were presented as unstandardized regression coefficients (B) along with standard errors and standardized regression coefficients (β), given the variables’ differing measurement scales. Additionally, to minimize potential reverse causality and further confirm the reliability of the observed associations, we repeated the mediation analysis after excluding participants who had cognitive impairment at baseline. All statistical procedures were performed using R software (version 4.4.1), with statistical significance set at a two-tailed p-value threshold of 0.05.

### 2.4 Ethics approval

Ethical approval for the CHARLS study was granted by the Ethics Committee at Peking University, and informed consent in written form was acquired from each participant. No additional ethical approval was required for this study.

## 3. Results

### 3.1 Characteristics of participants

The baseline characteristics of this study sample are presented in Table 1. The total sample included 2,320 older adults with a mean age of 66.2 years (SD = 5.2); males comprised 57.8% of the sample. Approximately 66.3% of participants had an education level of primary school or below, and approximately 56.5% resided in rural areas. Nearly half of the individuals (50.4%) reported having two or more chronic conditions. In terms of BMI, 62% fell within the normal weight range, while 33.5% were classified as overweight. At baseline, the average PEF was recorded at 328.8 L/min (SD = 116.5), and the mean score on the CES-D scale was 6.7 (SD = 5.7). The mean PEF for male and female participants was 373.7 L/min (SD = 117.6) and 267.2 L/min (SD = 81.5), respectively. The average cognitive function score at baseline was 12.5 (SD = 2.9), which declined to 11.8 (SD = 3.6) over the three-year follow-up period. Further details regarding participant characteristics throughout the follow-up phase are available in S1 Table.

**Table 1:** Characteristics of participants in 2015 and cognitive function in 2018.

Characteristics	Total (2,320) N (%)	Female (979) N (%)	Male (1,341) N (%)
Age (years), mean (SD)	66.2 (5.2)	65.8 (4.9)	66.5 (5.4)
The highest education level			
Illiterate	304 (13.1)	225 (23.0)	79 (5.9)
Primary school	1,235 (53.2)	517 (52.8)	718 (53.5)
Middle school	513 (22.1)	170 (17.4)	343 (25.6)
High school and above	268 (11.6)	67 (6.8)	201 (15.0)
Family residence			
Rural	1,310 (56.5)	506 (51.7)	804 (60.0)
Urban	1,010 (43.5)	473 (48.3)	537 (40.0)
Marital status			
Married	2,007 (86.5)	790 (80.7)	1,217 (90.8)
Single^a^	313 (13.5)	189 (19.3)	124 (9.2)
Number of chronic conditions			
0	472 (20.3)	164 (16.8)	308 (23.0)
1	679 (29.3)	249 (25.4)	430 (32.1)
≥ 2	1,169 (50.4)	566 (57.8)	603 (45.0)
BMI^b^ (kg/m²)			
Underweight (BMI < 18.5)	102 (4.4)	34 (3.5)	68 (5.1)
Normal (18.5 ≤ BMI < 25)	553 (56.5)	553 (56.5)	887 (66.1)
Overweight (25 ≤ BMI < 30)	666 (28.7)	325 (33.2)	341 (25.4)
Obesity (BMI ≥ 30)	112 (4.8)	67 (6.8)	45 (3.4)
Smoking status			
No	1,140 (49.1)	884 (90.3)	256 (19.1)
Yes	1,180 (50.9)	95 (9.7)	1,085 (80.9)
Drinking status			
No	1,110 (47.8)	745 (76.1)	365 (27.2)
Yes	1,210 (52.2)	234 (23.9)	976 (72.8)
Physical activity			
No	1,175 (50.6)	484 (49.4)	691 (51.5)
Yes	1,145 (49.4)	495 (50.6)	650 (48.5)
Total wealth (¥), mean (SD)	257,970.5 (115,898.2)	266,104.8 (133,180.7)	252,095.3 (101,679.7)
PEF^c^, mean (SD)	328.8 (116.5)	267.2 (81.5)	373.7 (117.6)
Depressive symptoms, mean (SD)	6.7 (5.7)	7.9 (6.2)	5.9 (5.2)
Cognitive function in 2015, mean (SD)	12.5 (2.9)	12.2 (3.2)	12.8 (2.7)
Cognitive function in 2018, mean (SD)	11.8 (3.6)	11.2 (4.0)	12.1 (3.3)

Single: never married, separated, and divorced; BMI = body mass index; PEF = peak expiratory flow.

### 3.2 Correlation between key variables

The results of Spearman correlation analysis indicated notable relationships among key variables. Pulmonary function was positively correlated with cognitive function (r = 0.20, P < 0.001), and negatively correlated with depressive symptoms (r = −0.15, P < 0.001). Additionally, higher levels of depressive symptoms were linked to lower cognitive function scores (r = −0.19, P < 0.001). Comprehensive correlation results can be found in Table 2.

**Table 2:** Correlations among PEF, depressive symptoms and cognitive function.

Variables	PEF	Depressive symptoms	Cognitive function
PEF	1.00		
Depressive symptoms	−0.15^***^	1.00	
Cognitive function	0.20^***^	−0.19^***^	1.00

*** P-value < 0.001, PEF = peak expiratory flow.

### 3.3 Mediating role of depressive symptoms in the relationship between baseline pulmonary function and follow-up cognitive function

As presented in S2 Table, after adjusting for control variables, PEF was significantly associated with depressive symptoms (β = −0.064, P < 0.001). In Table 3, Model 1 indicated a significant association between PEF and cognitive function (β = 0.131, P < 0.001). When depressive symptoms were included as a mediating variable in Model 2, the relationship between PEF and cognitive function remained significant (β = 0.122, P < 0.001). These findings suggest that depressive symptoms partially mediate the link between PEF and cognitive ability.

**Table 3:** Associations of PEF and depressive symptoms with subsequent cognitive function.

Variables	Model 1				Model 2			
	B	SE	β	P value	B	SE	β	P value
PEF	0.004	0.0008	0.131	<0.001	0.0038	0.0008	0.122	<0.001
Depressive symptoms					−0.085	0.013	−0.134	<0.001
Age	−0.036	0.017	−0.050	0.001	−0.040	0.017	−0.055	<0.001
Gender (reference = female)	−0.191	0.280	−0.257	0.494	−0.353	0.278	−0.047	0.204
Education (reference = High school and above)								
Illiterate	−5.011	0.355	−0.470	<0.001	−4.887	0.352	−0.459	<0.001
Primary school	−1.865	0.284	−0.253	<0.001	−1.698	0.221	−0.232	<0.001
Middle school	−0.218	0.307	−0.025	0.478	−0.209	0.303	−0.024	0.490
Family residence (reference = Rural)	0.828	0.111	0.175	<0.001	0.690	0.175	0.093	<0.001
Marital status (reference = Married)	−0.673	0.242	−0.065	0.005	−0.718	0.056	−0.234	<0.001
Number of chronic conditions (reference ≥2)								
0	−0.106	0.219	−0.016	0.629	−0.0 93	0.220	−0.108	0.652
1	0.009	0.001	−0.008	0.963	−0.115	0.193	−0.014	0.553
BMI (reference = Normal weight)								
Underweight	−0.604	0.421	−0.033	0.152	−0.407	0.515	−0.028	0.217
Overweight	0.186	0.152	0.023	0.081	0.239	0.324	0.030	0.434
Obesity	0.635	0.399	0.036	0.162	0.562	0.395	0.032	0.154
Physical activity (reference = No)	−0.004	0.166	0.006	0.803	−0.062	0.164	−0.008	0.703
Total wealth	6.3e-8	7.1e-8	0.020	0.375	8.2e-8	7.1e-8	0.259	0.244
Smoking status (reference = No)	−0.204	0.234	−0.277	0.383	−0.173	0.232	−0.024	0.454
Drinking status (reference = No)	−0.127	0.193	−0.017	0.509	−0.102	0.191	−0.014	0.591

B = unstandardized regression coefficient; SE = standard error; β = standardized regression coefficient. PEF = peak expiratory flow; BMI = body mass index.

**Fig 2:**
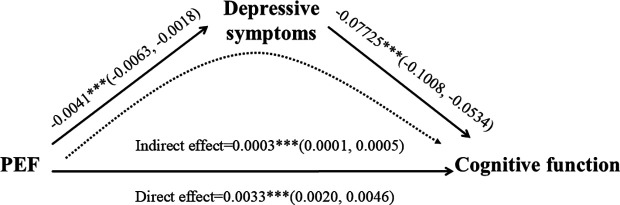
Path diagram of the mediating effect of depressive symptoms in the association between pulmonary function and cognitive function.

Additionally, results from the bootstrap analysis indicated that the total effect of baseline pulmonary function on cognitive outcomes at follow-up was 0.0036 (95% CI: 0.0023–0.0049). The indirect effect mediated by depressive symptoms was estimated at 0.0003 (95% CI: 0.0001–0.0005), accounting for approximately 9.1% (95% CI: 0.035–0.150) of the total effect. These outcomes underscore the important mediating role of depressive symptoms in the association between pulmonary health and cognitive functioning. A visual depiction of the mediation model is provided in Fig 2.

### 3.4 Sensitivity analyses

Among the 2,320 participants included in the analysis, 385 individuals whose cognitive function scores were more than one standard deviation below the mean were classified as having cognitive impairment (S3 Table). After excluding these participants and reanalyzing the data, the results remained consistent (S4 Table). The proportion of the effect of PEF on cognitive function mediated through depressive symptoms was 6.9% (95% CI: 0.026–0.140).

## 4. Discussion

This study used nationally representative data from CHARLS to systematically analyze the associations between pulmonary function, depressive symptoms, and cognitive function, and to test whether depressive symptoms mediate the pulmonary–cognitive relationship. We found that PEF correlated positively with cognitive function and inversely with depressive symptoms. Depressive symptoms mediated 9.1% of the total effect of PEF on cognitive function.

To elucidate the pathway linking pulmonary function and cognitive function, we applied the Baron–Kenny mediation steps for clarity and supplemented them with nonparametric bootstrap resampling (1,000 iterations) to estimate indirect effects [21,22]. This bootstrap approach does not require normality assumption, and therefore provides more robust confidence intervals, aligning our analysis with current best practices.

The findings of this study are consistent with previous research, indicating a significant association between decreased pulmonary function and cognitive decline [23]. Poor pulmonary function can cause chronic brain hypoxia, potentially leading to damage to brain cells and degeneration of brain tissue—all of which may contribute to cognitive impairment in older adults [24]. Chronic hypoxia is often accompanied by inflammatory responses [25], which can impact cognitive function through multiple pathways. On the one hand, this hypoxic-inflammatory state impairs cardiopulmonary function, leading to reduced cerebral perfusion [25]. On the other hand, it increases the risk of stroke and microvascular lesions, compromising brain integrity and function [26].

This study reinforces the mediating role of depressive symptoms in the relationship between pulmonary function and cognitive function. Reduced pulmonary function can give rise to various biological and psychosocial factors that increase the risk of depression. Chronic hypoxia caused by this decline may disrupt neurotransmitter balance and promote a chronic inflammatory response linked to depressive symptoms [27,28]. Such respiratory limitation frequently restricts physical activity and social engagement, potentially resulting in isolation and emotional stress. Over the long term, these overlapping influences increase susceptibility to depressive disorders in individuals with compromised pulmonary function [29–31]. In turn, depressive symptoms may further impair cognition through mechanisms such as chronic inflammation, neurotransmitter imbalance, and diminished neuroplasticity [32–34]. Therefore, depressive symptoms may serve as a critical bridge linking pulmonary function and cognitive function.

This study utilizes data from CHARLS, a nationally representative dataset with a large sample size, enhancing the generalizability of the findings. In the analysis, multivariable regression, bootstrap mediation, and sensitivity analyses were conducted. In the sensitivity analyses, we excluded participants with baseline cognitive impairment to confirm the stability of our findings, thus strengthening the overall robustness and reliability of our results.

Although PEF does not capture the full range of pulmonary function such as FEV1 and FVC, its simplicity makes it useful for large-scale studies [35]. It has been widely used for monitoring asthma and COPD, and it correlates well with FEV1 in moderate-to-severe airflow obstruction [36,37]. As this study relied only on PEF, future research should include additional spirometric measures to better assess lung function and its link to cognition.

This study has several limitations that need attention. First, although we used longitudinal data, we cannot completely rule out potential confounders or residual biases, such as genetic predispositions, unmeasured chronic conditions, or medication usage, which may have influenced the findings. Moreover, pulmonary function was measured with PEF, which offers only a rough estimate rather than the precise information that tests like forced expiratory volume in one second (FEV1) or forced vital capacity (FVC) could provide. Third, depressive symptoms were measured only at baseline, and we were unable to capture their potential fluctuations over time. This limitation may affect the interpretation of the mediation pathway, which assumes a degree of temporal stability in depressive symptoms. Fourth, the use of self-reported scales for assessing depressive symptoms and cognitive function may have introduced reporting bias and measurement errors. Finally, the study sample consisted exclusively of older Chinese adults. While this focus enhances the relevance of our findings to China’s rapidly aging population, it may limit the generalizability of the results to other age groups or ethnic populations. Future research is needed to replicate these findings in more diverse and international samples.

Despite these limitations, the findings of this study have important theoretical and clinical implications. Identifying the mediating role of depressive symptoms in the relationship between pulmonary function and cognitive function deepens our understanding of how pulmonary function decline affects cognitive function and provides a theoretical foundation for targeted health interventions in older adults. This study underscores the need for clinicians to monitor and assess depressive symptoms in older patients with declining pulmonary function, aiming to improve cognitive function and overall quality of life.

## 5. Conclusion

This study identified a significant association between pulmonary function and cognitive function among Chinese older adults, with depressive symptoms partially mediating this relationship. Regular pulmonary assessments, mental health screening, and targeted interventions are recommended to support cognitive health and promote healthy aging.

## Supporting information

S1 Table Characteristics of participants included in the final analysis. (CSV)

S2 Table Associations of PEF with depressive symptoms. (XLSX)

S3 Table Characteristics of participants excluding cognitive function impaired in sensitivity analysis. (CSV)

S4 Table Associations of PEF and depressive symptoms with cognitive function excluding cognitive function impaired in sensitivity analysis. (DOCX)
