# Supplementary material for: Mediation analysis of depressive symptoms in the relationship between pulmonary function (measured by peak expiratory flow) and cognitive function among older adults in Chinese
Source: PLoS One. 2025 Jul 24;20(7):e0328231. doi: 10.1371/journal.pone.0328231 (PMC12289082; doi:10.1371/journal.pone.0328231)
Supplement: S4 Table — (DOCX) [file pone.0328231.s004.docx]

**Supplementary Table S4. Associations of PEF and depressive symptoms with subsequent cognitive function excluding cognitively impaired participants.**

| Variables | Model 1 | | | | Model 2 | | | |
| --- | --- | --- | --- | --- | --- | --- | --- | --- |
|  | B | SE | β | P value | B | SE | β | P value |
| PEF | 0.0038 | 0.001 | 0.135 | <0.001 | 0.0035 | 0.0008 | 0.126 | <0.001 |
| Depression |  |  |  |  | -0.068 | 0.016 | -0.113 | <0.001 |
| Age | -0.140 | 0.018 | -0.021 | 0.447 | -0.018 | 0.018 | -0.027 | 0.328 |
| Gender (reference = female) | -0.298 | 0.289 | -0.044 | 0.301 | -0.422 | 0.288 | -0.062 | 0.144 |
| Education (reference = High school and above) |  |  |  |  |  |  |  |  |
| Illiterate | -3.946 | 0.376 | -0.357 | <0.001 | -3.861 | 0.374 | -0.349 | <0.001 |
| Primary school | -1.609 | 0.274 | -0.243 | <0.001 | -1.537 | 0.273 | -0.232 | <0.001 |
| Middle school | -1.156 | 0.294 | -0.015 | 0.694 | -0.092 | 0.292 | -0.012 | 0.752 |
| Family residence (reference = Rural) | 0.746 | 0.180 | 0.112 | <0.001 | 0.660 | 0.180 | 0.099 | <0.001 |
| Marital status (reference = Married) | -0.876 | 0.253 | -0.092 | <0.001 | -0.807 | 0.252 | -0.085 | 0.001 |
| Number of chronic conditions (reference ≥2 ) |  |  |  |  |  |  |  |  |
| 0 | 0.289 | 0.226 | 0.035 | 0.205 | 0.133 | 0.228 | 0.016 | 0.559 |
| 1 | 0.057 | 0.200 | 0.008 | 0.775 | -0.037 | 0.200 | -0.005 | 0.854 |
| BMI (reference = Normal weight) |  |  |  |  |  |  |  |  |
| Underweight | -0.626 | 0.464 | -0.035 | 0.177 | -0.595 | 0.461 | -0.033 | 0.197 |
| Overweight | 0.194 | 0.193 | 0.027 | 0.315 | 0.169 | 0.192 | 0.023 | 0.378 |
| Obesity | 0.471 | 0.401 | 0.031 | 0.240 | 0.411 | 0.398 | 0.027 | 0.302 |
| Physical activity (reference = No ) | 0.003 | 0.170 | 0.004 | 0.988 | -0.018 | 0.169 | -0.003 | 0.914 |
| Total wealth | 1.28e-7 | 7.47e-8 | 0.044 | 0.085 | 1.39e-7 | 7.41e-8 | 0.048 | 0.061 |
| Smoking status (reference = No) | -0.332 | 0.240 | -0.050 | 0.204 | -0.301 | 0.238 | -0.045 | 0.207 |
| Drinking status (reference = No) | -0.254 | 0.200 | -0.038 | 0.134 | -0.238 | 0.199 | -0.036 | 0.231 |

B = unstandardized regression coefficient; SE = standard error; β = standardized regression coefficient. PEF= peak expiratory flow；BMI= body mass index.
